# Prognostic and tumor microenvironmental feature of clear cell renal cell carcinoma revealed by m6A and lactylation modification-related genes

**DOI:** 10.3389/fimmu.2023.1225023

**Published:** 2023-08-11

**Authors:** Lin Yang, Xiaoyu Wang, Jiahao Liu, Xiaoqiang Liu, Sheng Li, Fuchun Zheng, Qianxi Dong, Songhui Xu, Jing Xiong, Bin Fu

**Affiliations:** Department of Urology, First Affiliated Hospital of Nanchang University, Nanchang, China

**Keywords:** lactylation, M6A, clear cell renal cell carcinoma, immunotherapy, tumor microenvironment

## Abstract

**Background:**

Both lactylation and m6A modification have important implications for the development of clear cell renal cell carcinoma (ccRCC), and we aimed to use crosstalk genes of both to reveal the prognostic and immunological features of ccRCC.

**Methods:**

Our first step was to look for lactylation-related genes that differed between normal and tumor tissues, and then by correlation analysis, we found the genes associated with M6A. Following that, ccRCC subtypes will be identified and risk models will be constructed to compare the prognosis and tumor microenvironment among different subgroups. A nomogram was constructed to predict the prognosis of ccRCC, and *in vitro*, experiments were conducted to validate the expression and function of key genes.

**Results:**

We screened 100 crosstalk genes and identified 2 ccRCC subtypes. A total of 11 prognostic genes were screened for building a risk model. we observed higher immune scores, elevated tumor mutational burden, and microsatellite instability scores in the high-risk group. Therefore, individuals classified as high-risk would derive greater benefits from immunotherapy. The nomogram’s ability to predict overall survival with a 1-year AUC of 0.863 demonstrates its significant practical utility. In addition, HIBCH was identified as a potential therapeutic target and its expression and function were verified by *in vitro* experiments.

**Conclusion:**

In addition to developing a precise prognostic nomogram for patients with ccRCC, our study also discovered the potential of HIBCH as a biomarker for the disease.

## Background

Renal cell carcinoma (RCC), particularly clear cell cancers which account for approximately 80% of cases, are highly aggressive and heterogeneous tumors (1). Due to the lack of specific symptoms in early RCC, nearly 30% of patients present with distant metastases at the time of initial diagnosis, which is one of the major reasons for the poor prognosis of RCC patients (2, 3). An excellent outcome is usually achieved by surgically resecting the primary lesion of a low-risk limited clear cell renal cell carcinoma (ccRCC), but a significant proportion of patients still recur within a short period (approximately 40%). Patients with high-risk metastatic or limited ccRCC must undergo systemic therapy to improve their prognosis (4, 5). In recent years, combination therapies based on anti-angiogenic agents and immune checkpoint inhibitors have been shown to improve the survival of ccRCC patients (6). Nevertheless, the current problem is that effective long-term treatment responses can only be observed in a small number of patients (7, 8). In the era of precision medicine, it is crucial to thoroughly understand the tumor microenvironment (TME) and identify biomarkers associated with therapeutic response to effectively manage ccRCC patients in the long term.

Aerobic glycolysis is an important feature of tumor cell energy metabolism known as the “Warburg effect”, which leads to a large accumulation of lactic acid in the TME (9). Recent findings suggest that lactate in TME can regulate immune cell metabolism through mitochondrial metabolic pathways, thereby affecting immune surveillance and escape-related behaviors (10, 11). A study by Zhao et al. proposed a novel epigenetic modification that translates the cellular metabolic state into a stable gene expression pattern through histone lactylation modification (12). This provides a new direction for understanding the mechanisms by which lactate regulates cellular metabolism and immune function. Currently, it has been demonstrated that lactylation plays a key role in the progression of ccRCC. Yang et al. found that Inactive von Hippel-Lindau-triggered (VHL) histone lactylation can drive the progression of ccRCC (13). More interestingly, Yu and Xiong et al. found that histone lactylation drives N6-adenylation methylation modifications (m6A) to promote tumor progression and immunosuppression (14, 15). Moreover, ccRCC progression and immune landscapes are strongly influenced by m6A modification (16). However, the impact of crosstalk between histone lactylation and m6A modification on the ccRCC TME is unclear.

Therefore, we utilized the interaction between histone lactylation and m6A modification-related genes to forecast patient survival and assess the response to immunotherapy in ccRCC.

## Methods

### Datasets

A training set of TCGA-KIRC data was downloaded from the Cancer Genome Atlas (TCGA) database, which contained gene expression data from 541 tumor tissues and 72 normal tissue samples, as well as corresponding clinical information. The E-MTAB-1980 validation set, which contains clinical and gene expression data from 101 patients with ccRCC, was generated from ArrayExpress. Our next step was to remove genes with raw counts below 10 in more than 25% of the samples. The TPM data were transformed into log2 (TPM+1). There are 1223 genes associated with lactylation modification according to Zhang et al. (12).

### Correlation and difference analysis

The 1223 lactylation modification-related genes were subjected to Pearson correlation analysis with 23 m6A genes to obtain crosstalk genes with screening criteria of correlation>0.5 and padj<0.01. To identify differentially expressed genes (DEGs) between cancer and paracancerous tissues, we used the “DESeq2” package [padj <0.05, |log2fold change (FC)|>1]. Subsequently, the crosstalk genes were merged with DEGs and the intersection was taken to finally obtain the differentially expressed crosstalk genes (DECGs). The correlation results between DECGs were visualized using the “circlize” package.

Each sample’s tumor mutation load (TMB) was calculated using the “Maftools” package. Gene Ontology (GO) and Kyoto Encyclopedia of Genes and Genomes (KEGG) analyses of DECGs were performed using the “clusterProfiler” package.

### Identification of ccRCC subtypes

We identified ccRCC clinical subtypes using the consensus clustering R package “ConsensusClusterPlus” (17). The ability of DECGs to discriminate between subtypes was assessed using principal component analysis (PCA). After that, we compared the differences between subtypes in terms of clinical variables (age, gender, grade, and stage) as well as overall survival (OS).

### The immune landscape of different subtypes

Every sample was analyzed for the TME score using the “estimate” package. In each TCGA-KIRC sample, immune cell infiltration was assessed using the online analysis tool TIMER2.0 (CIBERSORT algorithm). Based on our knowledge of the close relationship between immune-inhibitory, immune-stimulatory, and human leukocyte antigen (HLA) genes and TME, a comparison of expression levels between subtypes was made (18, 19).

### Constructing risk model

Univariate COX regression analysis was first performed on the DECGs to screen for genes associated with OS according to p<0.05, and they were used to perform a least absolute shrinkage and selection operation (LASSO) analysis to screen for genes most associated with prognosis for constructing the risk model and to derive a risk coefficient for each gene. The expression of each modeled gene was multiplied by the risk coefficient to calculate the risk score for each patient. Each group of patients was categorized according to the median risk score. To test whether the model was able to discriminate between patients at different risks, PCA and Kaplan-Meier (K-M) survival analyses were performed. In addition, the relationship between clinical variables and risk scores for different clinical characteristics in the high- and low-risk groups was assessed.

### Immune landscape and enrichment analysis

Based on the above results, we evaluated the differences between the two risk groups in terms of TME scores, immune cell infiltration, and immune-related gene expression. Then, we calculated immune-related function scores using the “ GSEABase” and “GSVA” packages based on the “ immune. gmt” file. The DEGs were determined using the “limma” package, followed by GO, KEGG, and gene set enrichment analysis (GSEA) using “ClusterProfiler”. The “enrichplot” and “GseaVis” packages were used to visualize the enrichment analysis results.

As a result of the analysis above, we obtained the sample TMB and then downloaded the MSI score file *via* the “cBioPortalData” package. We defined samples as MSI when the score exceeded 0.3, and MSS if the score was below it. Moreover, the “easier” package calculates an immunotherapy response score based on TME characteristics, where higher scores indicate greater immunotherapy sensitivity (20). We downloaded each patient’s immunophenotype score (IPS) from the Cancer Immunome Atlas (TCIA, https://tcia.at/home) and divided it into <=8 and >8 groups according to IPS. The relationship between these metrics and risk scores was finally evaluated to reflect the predictive value of risk scores on immunotherapy response.

### Constructing a nomogram

Through multivariate and univariate COX regressions, several independent predictors of OS were identified. A nomogram was then created with the help of the “survival” and “rms” packages. Calibration plots, Receiver Operating Characteristics (ROC), and Area Under Curve (AUC) were used to assess the predictive capability of the nomogram. Decision curve analysis (DCA) was used to determine the clinical value of the nomogram.

### Comprehensive analysis of key genes

Both the metabolism of lactate and the lactylation modification process are closely related to the function of mitochondria. We obtained 1136 mitochondria genes (MTGs) from the MitoCarta 3.0 database (https://www.broadinstitute.org/) and then took intersections with risk model genes to obtain key genes. Following the pan-cancer analysis, key genes were assessed for differential expression and prognostic value across multiple cancer types. An assessment of the association between clinical features and TME of ccRCC was conducted using the TISIDB database (http://cis.hku.hk/TISIDB/). The ChEA3 database (https://maayanlab.cloud/chea3/#top) was used to obtain potential TF regulating key genes, and we selected the “Mean Rank” panel and took the top 10 genes for subsequent analysis and further screened the TF regulating key genes by differential analysis and K-M survival analysis. Patients with ccRCC were categorized into two groups according to the expression of key genes. After performing a differential analysis using the “limma” package, downstream pathways were identified using GO, KEGG, and GSEA analyses.

### Cell culture, transfection, and infection

Cell lines used in this study were obtained from Procell Life Science&Technology Co., Ltd (Wuhan, China). An incubator containing 37°C and 5% CO2 was used to grow HK-2 and ACHN cells. Medium: MEM + 10% fetal bovine serum (FBS) + 1% antibiotics (HK-2 and ACHN), 1640 + 10% FBS + 1% antibiotics (786-O).

The overexpression plasmids and control plasmids of HIBCH were synthesized by Obio Technology (Shanghai) Corp., Ltd. We transfected the plasmid into 293T cells using calcium phosphate transfection to collect the viral fluid, which was then used to infect ACHN and 786-O cells, resulting in elevated levels of HIBCH expression in the cells. The RNA extraction was performed 48 hours after cell infection, and PCR was carried out to determine overexpression efficiency. Meanwhile, further cell phenotyping experiments were carried out.

### Quantitative PCR

For RNA extraction, we used TRIzol reagent (Invitrogen, Thermo Fisher Scientific, Inc.) (Eight pairs of ccRCC cancer and paracancerous tissue specimens were obtained from the Human Genetic Resources Center, The First Affiliated Hospital of Nanchang University.), and then reverse transcribed by Takara PrimeScript RT kit (Takara Bio, Inc., Otsu, Japan). The qPCR was performed on a Roche LightCycler96 real-time fluorescent quantitative PCR system using an SYBR premix Ex Taq kit (Takara Bio, Inc., Otsu, Japan). The relative expression of genes was calculated based on the 2^-ΔΔCt method. Primer sequences: HIBCH-F: 5’-GGAGTTGGTCTCTCAGTCCATG-3’, HIBCH-R: 5’-CCAAGTTTTCCTTGGAGTCGTGG-3’.

### Cell migration

Cell migration was measured using 24-well transwell chambers, each upper chamber was inoculated with approximately 30,000 cells, and 200ul of FBS-free medium was added; the lower chamber was filled with 600ul of medium containing 20% FBS and counted after 36 hours. Cells were cultured to 80% density in 6-well plates, scratched, and then switched to an FBS-free medium and photographed in the same field of view at 0 h and 24 h, respectively.

### Statistical analysis

Statistical analyses were conducted using R (version 4.2.2) or GraphPad Prism (version 9.0), and p<0.05 was considered statistically significant. Analysis of variance was used to compare categorical variables, and t-tests were used to compare continuous variables. Correlations between continuous variables were examined by Spearman or Pearson correlation analysis. Non-parametric samples comparing two independent samples were compared using Wilcoxon, while multiple independent samples were compared using Kruskal-Wallis.

## Results

### Screening and analysis of DECGs

**Figure 1:**
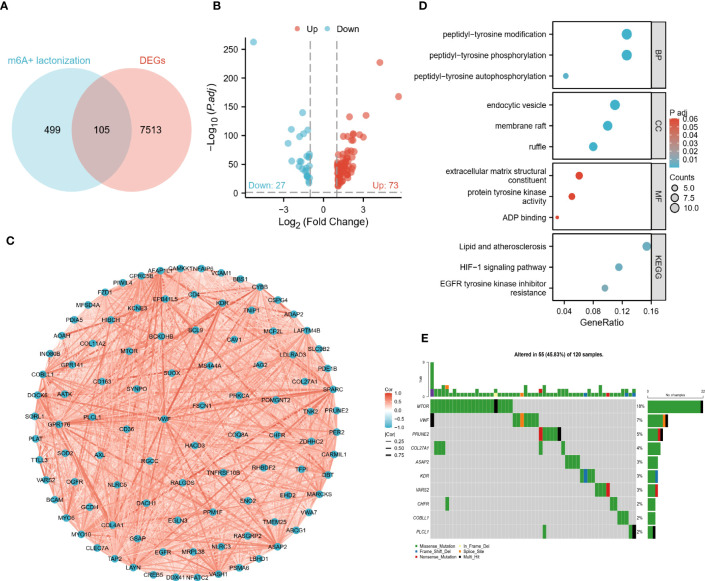
Identification and preliminary analysis of DECGs. **(A)** Venn diagram of crosstalk genes and DEGs intersected. **(B)** Volcano plots show the differential distribution of DECGs. **(C)** Correlation network diagram of DECGs. **(D)** DECGs are enriched for biological functions and BP, Biological Process; MF, Molecular function; CC, Cellular component). **(E)** Mutational landscape of DECGs.

By correlation analysis, we obtained 604 crosstalk genes, including 105 DEGs (**Figure 1A**). Then we removed 5 genes that were not detected in the validation cohort and finally obtained 100 DECGs. As shown in the volcano plot, there were 27 low and 73 high-expressed genes in the tumor tissue (**Figure 1B**). Subsequently, correlation network plots demonstrated a close association between DECGs (**Figure 1C**). the results of GO and KEGG analysis suggested that DECGs may be involved in biological processes such as protein modification and energy metabolism, and may play an important role in the HIF-1 signaling pathway (**Figure 1D**). Interestingly, the VHL/HIF pathway is linked to lactate production in ccRCC (13), which certainly suggests to us that these DECGs deserve to be studied in depth. According to **Figure 1E**, DECGs are mutated in 45.83% of samples, with MTOR showing the highest mutation frequency (18%).

### Clinical features of the two ccRCC subtypes

**Figure 2:**
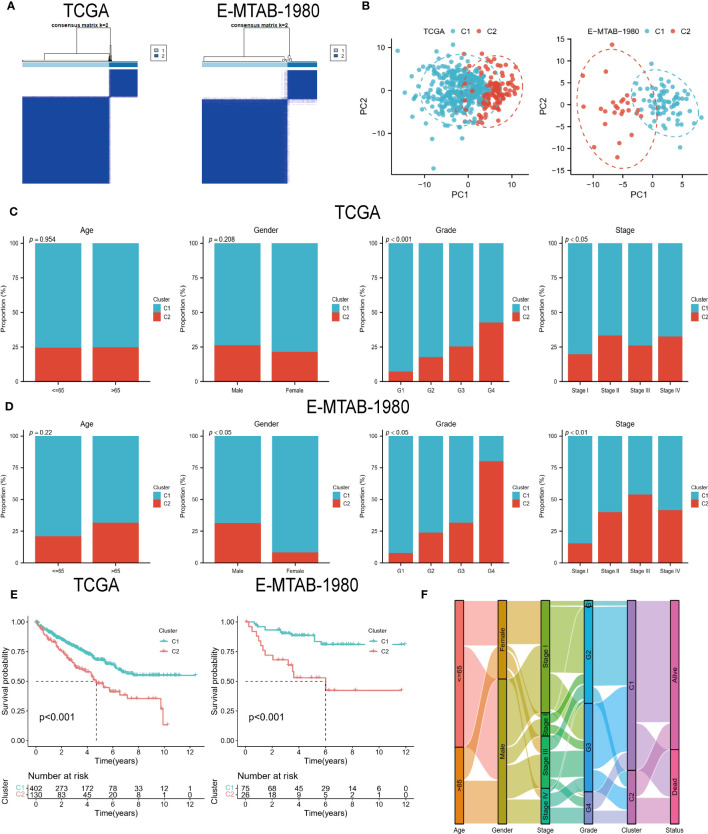
DECGs-based ccRCC subtypes. **(A)** Consensus clustering identified 2 ccRCC subtypes. **(B)** Principal component analysis based on DECGs. **(C, D)** Distribution of the 2 subtypes across clinical variables in the TCGA and E-MTAB-1980 cohorts. **(E)** K-M analysis to compare survival differences between the 2 subtypes. **(F)** Sankey diagrams show the interchangeable relationships between clinical variables, subtypes, and survival status.

Two subtypes of ccRCC were identified (**Figure 2A**), and the PCA confirmed this (**Figure 2B**). A comparison of the clinical characteristics of the two subtypes was conducted following that. In the TCGA cohort, age and gender did not differ between the two groups, whereas the distribution of grading and stage showed significant differences, with the C2 group having a higher nuclear grade and a more advanced clinical stage (**Figure 2C**). Although the E-MTAB-1980 cohort also showed the same trend, the C2 group in the cohort had more male patients (**Figure 2D**). The C2 group suffered a worse prognosis in both cohorts according to the K-M survival analysis (**Figure 2E**). In addition, the Sankey diagram more visually demonstrates the close association between subtypes and clinical features (**Figure 2F**).

### Two subtypes of the immune landscape

**Figure 3:**
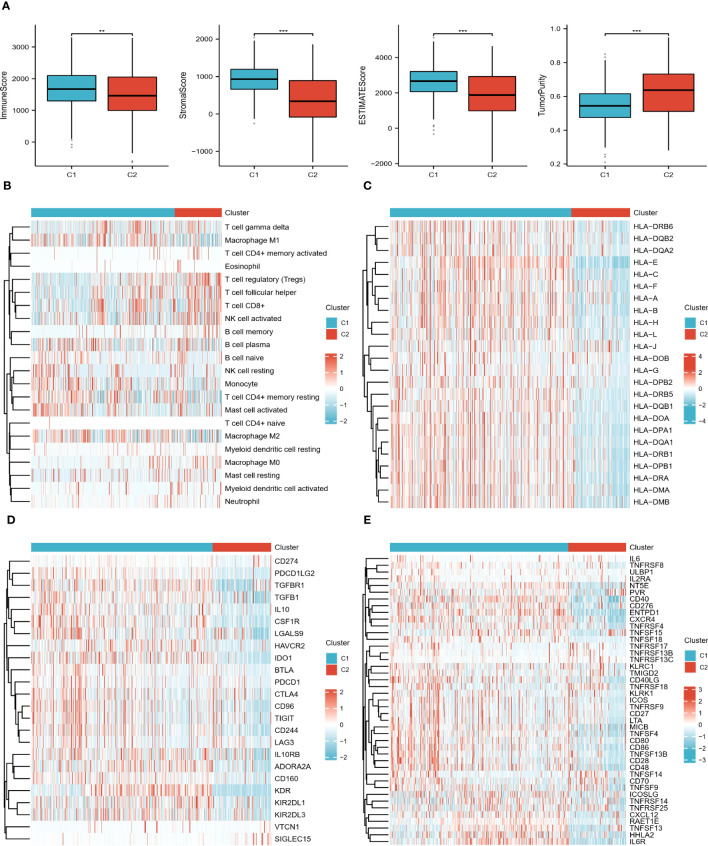
Immune landscape of the 2 subtypes. **(A)** Differences in TME scores between C1 and C2 groups. **(B)** Heat map of the distribution of 22 immune cells in the two subtypes. **(C–E)** Heat map of the difference in expression levels of HLA genes, immunoinhibitory, and immunostimulatory in the two subtypes. "**" <0.01, and "***" <0.001.

The results showed that all scores, except for tumor purity, showed higher levels in the C2 group (**Figure 3A**). **Figure 3B** shows that the two subtypes infiltrated differently with immune cells, for example, the C1 group had more abundant monocytes and macrophages; the C2 group was infiltrated with more T cell follicular helper (TFH), T cell regulatory (Tregs), and NK cell activated. In addition, it is clear from the heat map that most of the immunoinhibitory, immunostimulatory, and HLA genes were expressed at higher levels in the C1 group (**Figures 3C–E**). In conclusion, all of the above results indicate that the ccRCC subtypes identified by DECGs have distinct TME and clinical characteristics. Therefore, an in-depth analysis of the predictive value of DECGs for the prognosis and immunotherapy of ccRCC is warranted.

### Prognostic risk characteristics of ccRCC patients

**Figure 4:**
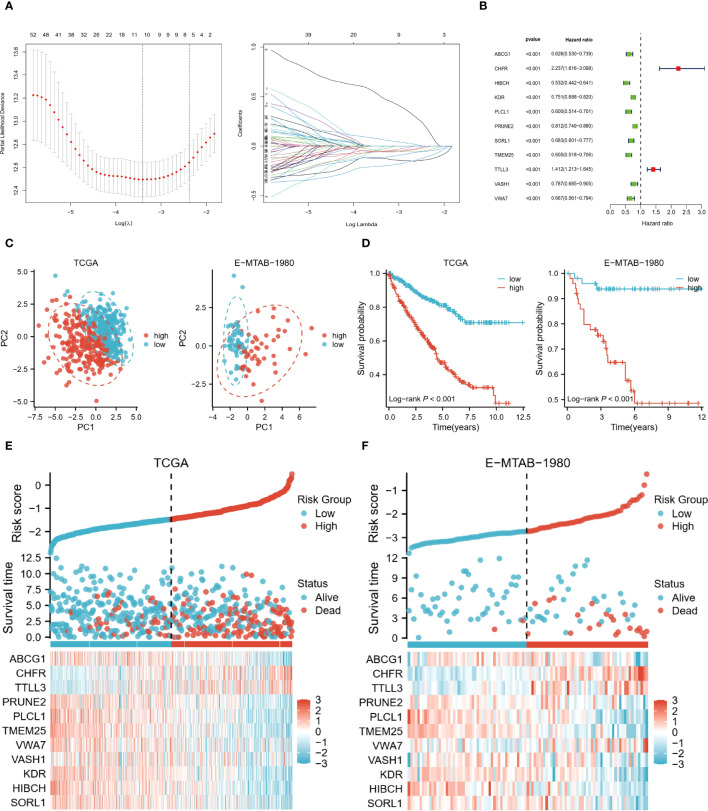
Risk model based on 11 genes. **(A)** LASSO regression analysis was performed to identify the genes used to construct the risk model. **(B)** Hazard ratio of the 11 model genes. **(C)** Principal component analysis based on model genes. **(D)** K-M analysis comparing OS of high and low-risk groups. **(E, F)** Trends in the distribution of survival status and model gene expression levels with changes in risk scores.

For the risk model, LASSO regression analysis identified 11 prognostic genes in the training set (**Figure 4A**). All were associated with a better prognosis, except TTLL3 and CHFR (**Figure 4B**). Risk score per patient = SORL1*(-0.056) + HIBCH*(-0.122) + KDR*(-0.047) + VASH1*(-0.016) + VWA7*(-0.036) + TMEM25*(-0.202) + PLCL1*(-0.116) + PRUNE2*(-0.055) + TTLL3*0.069+CHFR*0.501+ABCG1*(-0.054). According to PCA, these 11 risk genes (RGs) could be assigned to different risk groups of ccRCC patients (**Figure 4C**). K-M survival curves show that the high-risk group has shorter long-term survival times (**Figure 4D**). The validation cohort also demonstrated similar results. **Figures 4E, F** demonstrate the distribution of survival status and RGs with a risk score, which is almost consistent with the trend in both cohorts.

**Figure 5:**
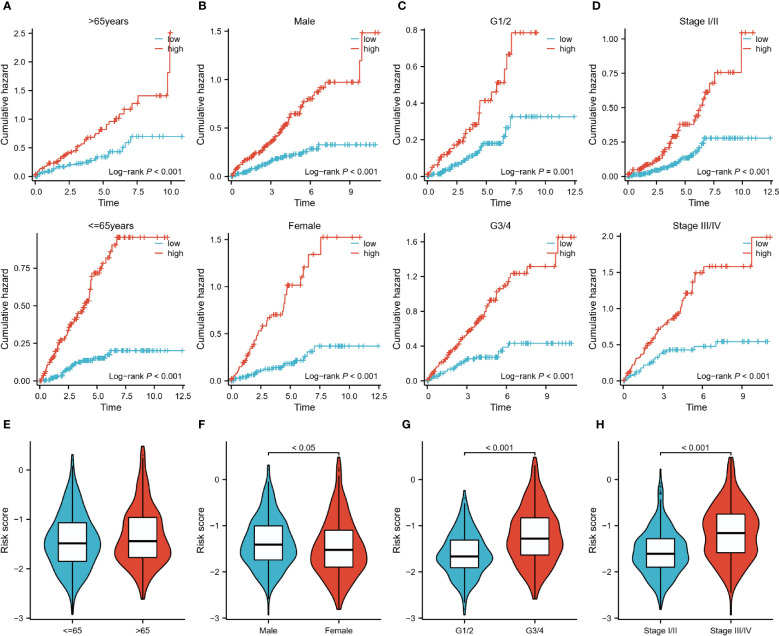
Risk characteristics for each subgroup of clinical variables. **(A–D)**: Cumulative hazard over time for <=65 and >65 years of age, male and female, G1/2 and G3/4, and Stage I/II and III/IV patients in high and low-risk groups. **(E–H)**: Differences in risk scores between patients <=65 years and >65 years, male and female patients, G1/2 and G3/4 patients, StageI/II and StageIII/IV.

Subsequently, we analyzed the risk characteristics of the different clinical variables. Among patients of different ages (<=65 vs >65), gender (Male vs Female), grades (G1/2 vs G3/4), and stages (Stage I/II vs Stage III/IV), the cumulative risk was increasing year by year in the high-risk group and was consistently higher than in the low-risk group (**Figures 5A–D**). We also found large differences in risk scores between variables within each variable. Although risk scores did not differ significantly between the two age groups, male, high-core graded (G3/4), and late-stage (Stage III/IV) patients tended to have higher risk scores (**Figures 5E–H**). In the Sankey diagram depicting the correlation between C1/C2 subtypes and risk groupings, we can observe that the C2 group with a worse prognosis is almost exclusively distributed in the high-risk group, whereas the C1 group, with a slightly better prognosis, is in a homogeneous distribution in the two risk groups (SF1 I).

### Immunological characterization and enrichment analysis of the two risk groups

**Figure 6:**
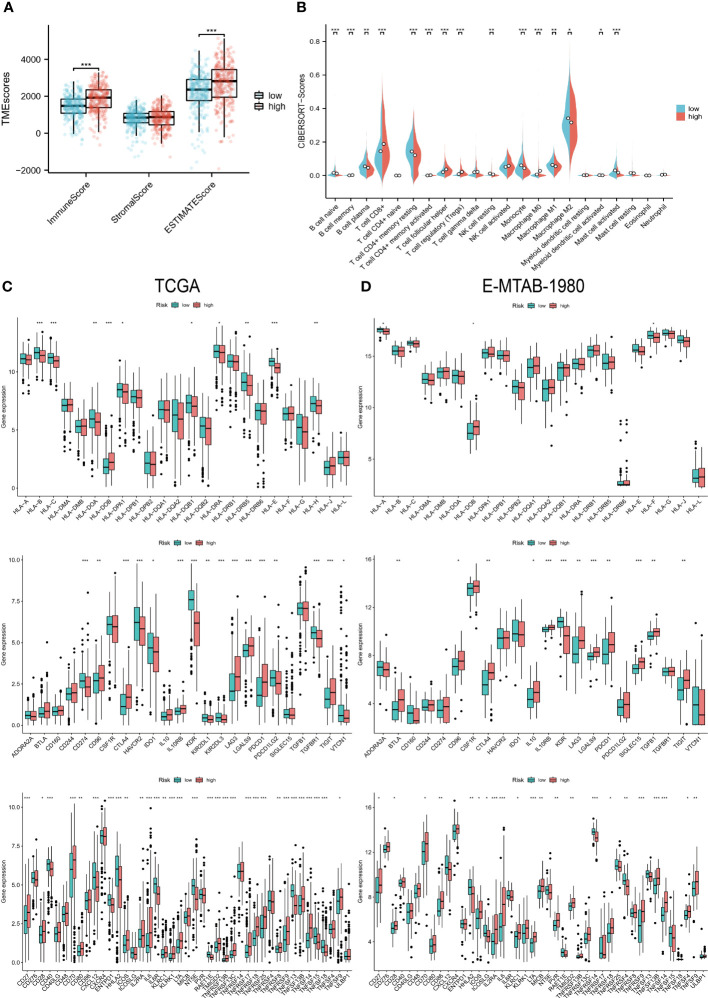
Immunological characteristics of the two risk groups. **(A)** Differences in TME scores between the high and low-risk groups. **(B)** Levels of infiltration of 22 immune cell types in the two risk groups. **(C, D)** HLA gene, immunoinhibitory, and immunostimulatory expression levels in the TCGA and E-MTAB-1980 cohorts in the high- and low-risk groups. “*” <0.5, "**" <0.01, and "***" <0.001.

In the TME score, both the immune score and the estimated score were higher in the high-risk group (**Figure 6A**). And the higher abundance of CD8 T cells and TFH clustered in the high-risk group (**Figure 6B**), which usually exerts anti-tumor immune effects. Additionally, only a few HLA genes showed differential expression, but most of the immunoinhibitory and immunostimulatory were different from them. High-risk patients expressed more CD96, CTLA4, IL10RB, LAG3, LGALS9, PDCD1, and TIGIT levels among immunoinhibitory molecules. The same is true for immunostimulatory, most of which are highly expressed in high-wind samples, such as CD70, IL6, and TNFRSF18 (**Figure 6C**). A similar expression pattern was observed in cohort E-MTAB-1980 (**Figure 6D**).

**Figure 7:**
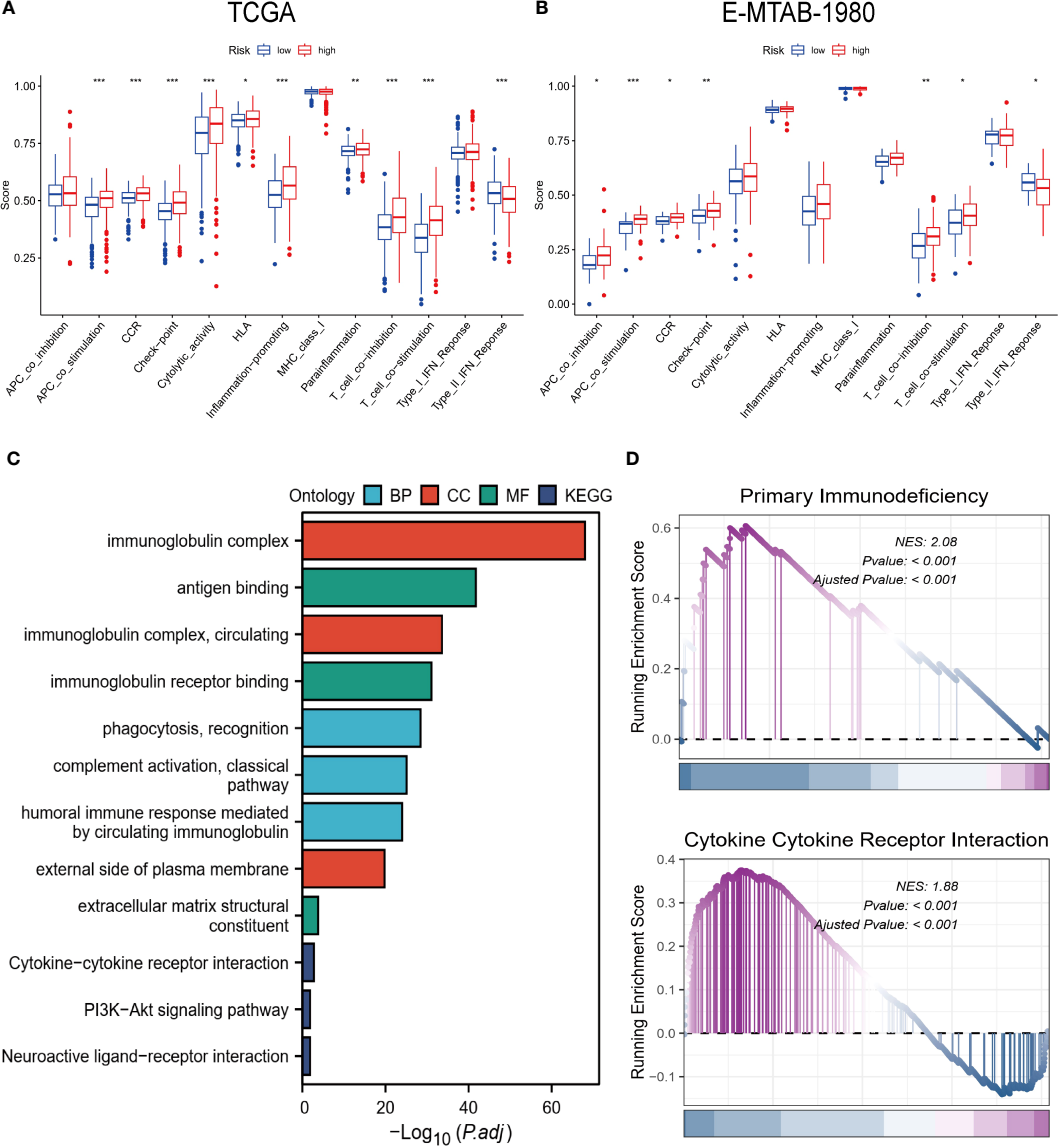
Biofunctional and pathway analysis. **(A, B)** Comparison of immune-related functions between high and low-risk groups. **(C)** Results of GO and KEGG enrichment analysis. **(D)** Visualization results of GSEA. “*” <0.5, "**" <0.01, and "***" <0.001.

The immune-related function scores were generally higher in the high-risk group than in the other group (**Figures 7A, B**). Furthermore, the results of GO, KEGG, and GSEA also showed a strong association of risk grouping with immune-related functions. Biological pathways and functions related to immunity were enriched in DEGs between high- and low-risk groups (**Figure 7B**). Moreover, primary immunodeficiency and cytokine-cytokine receptor interaction pathways were enriched in the high-risk group (**Figure 7C**). The series of results suggest a close association between risk groups and the immune microenvironment, especially in high-risk groups.

### Prediction of immunotherapy response

**Figure 8:**
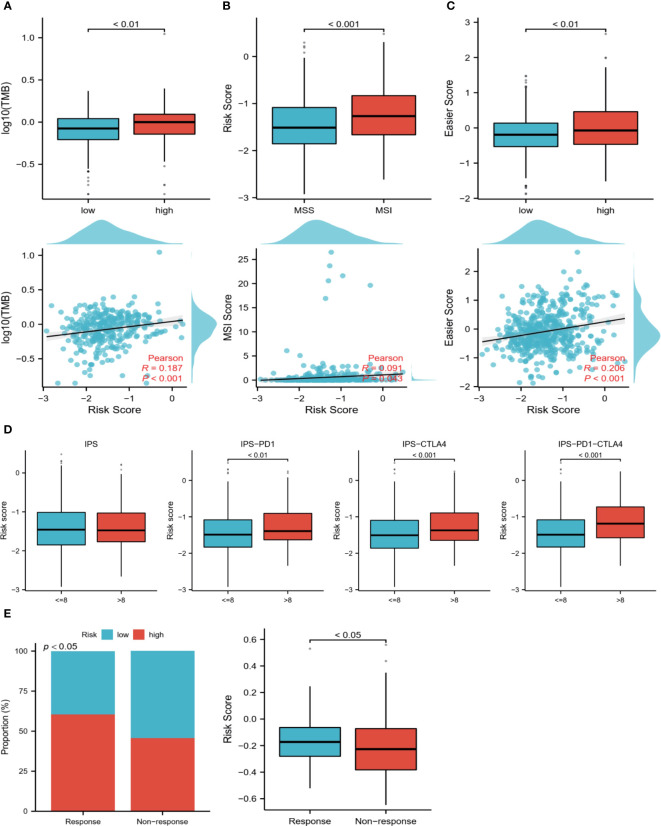
Prediction of the immune response. **(A)** TMB levels in high and low-risk groups, Pearson correlation analysis of TMB and risk scores. **(B)** Differences in risk scores between MSI and MSS groups, Pearson correlation analysis of risk scores with MSI scores. **(C)** Differences in easier scores in high and low-risk groups, and Pearson correlation analysis with risk scores. **(D)** The relationship between IPS grouping and risk scores. **(E)** Relationship between immunotherapy response and risk scores in a bladder cancer immunotherapy cohort.

Patients with ccRCC were analyzed based on TMB, MSI, IPS, and easier scores to predict their response to immunotherapy. High-risk samples showed significantly higher levels of TMB and easier scores, and the samples in the MSI group had higher risk scores (**Figures 8A–C**). Similarly, higher scores in the IPS scores about immune checkpoints were associated with higher risk scores (**Figure 8D**). According to the above results, TME differs greatly between the two risk groups, and the immunotherapy response may be more durable and effective in patients at high risk. Interestingly, we got the same results in a bladder cancer immunotherapy cohort. We found that patients in the immune-responsive group had higher risk scores and more high-risk patients (**Figure 8E**).

### Nomogram accurately predicts survival of ccRCC patients

**Figure 9:**
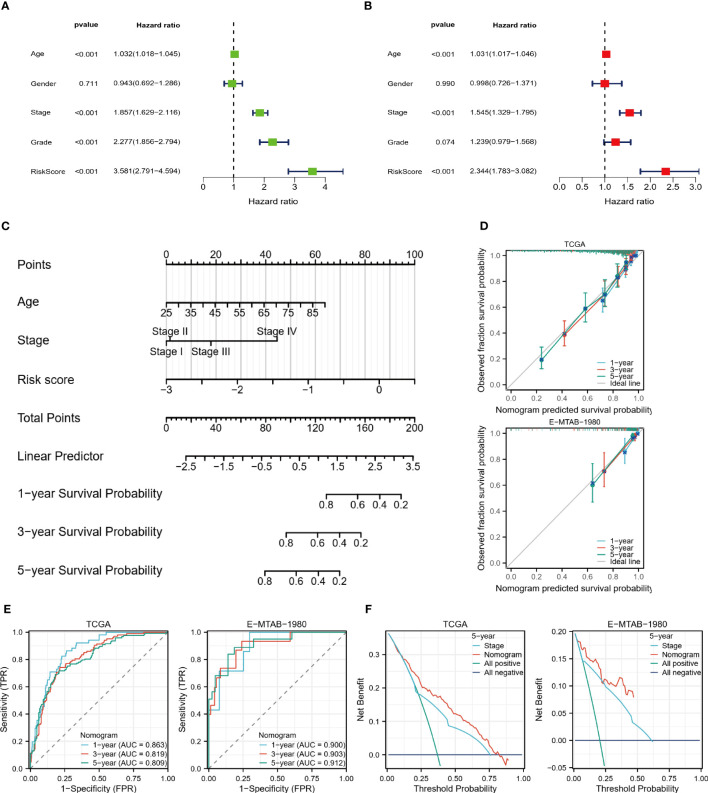
Nomogram predicts survival probability. **(A, B)** Univariate and multivariate COX regression analysis. **(C)** Nomogram constructed based on TCGA cohort. **(D)** Nomogram calibration plots for 1, 3, and 5 years. **(E)** 1, 3, and 5-year ROC curves and ACU to assess the predictive performance of the model. **(F)** 5-year DCA curves to assess the clinical application value of the model.

Using the training set, we performed univariate and multivariate COX regression analysis to identify independent predictors of OS, including age, stage, and risk score (**Figures 9A, B**). Based on the sum of the corresponding scores for each factor, a nomogram was constructed to predict patient survival at 1, 3, and 5 years (**Figure 9C**). Both the training and validation data sets showed the predicted probabilities to be almost in line with the actual probabilities (**Figure 9D**). Furthermore, the results of the ROC analysis also showed strong predictive performance of the model with 1-year AUC=0.863 for the TCGA cohort and 1-year AUC=0.900 for the validation cohort (**Figure 9E**). The DCA demonstrated that the nomogram was superior to the TNM staging for clinical purposes (**Figure 9F**).

### The role of the key gene HIBCH in ccRCC

**Figure 10:**
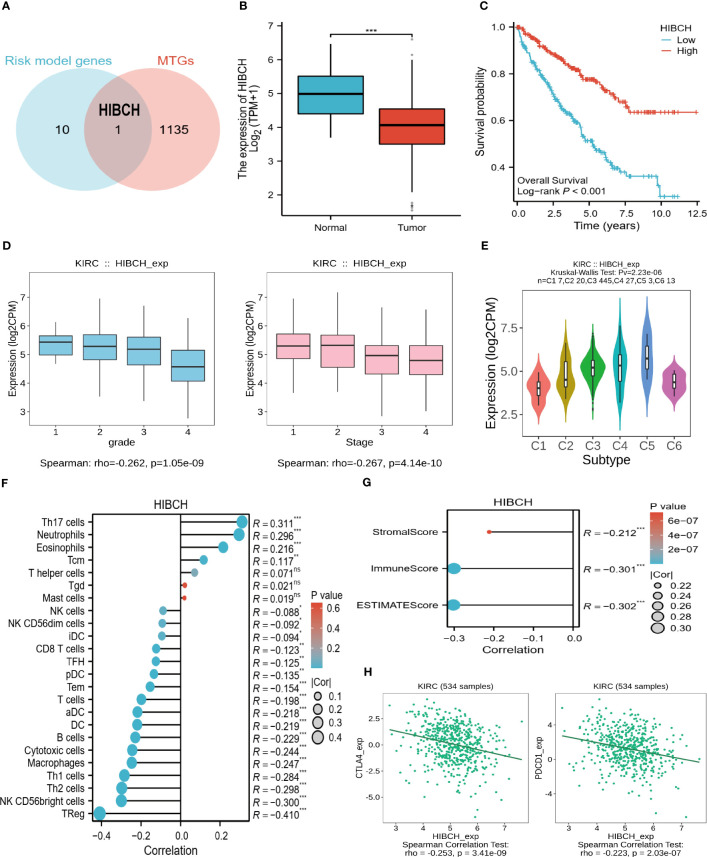
The significance of HIBCH in ccRCC. **(A)** The Venn diagram identified the key gene HIBCH. **(B)** HIBCH expression levels in tumor and normal tissues. **(C)** K-M survival curves between high and low expression groups of HIBCH. **(D)** HIBCH expression levels in samples of different grades and stages. **(E)** HIBCH can classify ccRCC into 6 immune subtypes. **(F, G)** Lollipop plot of the correlation between HIBCH and immune cell infiltration level, TME score. **(H)** Correlation of HIBCH with the expression of CTLA4, PDCD1. "***" <0.001.

From risk model genes, we identified HIBCH, a gene closely related to the mitochondrial function that may play a role in the development of ccRCC (**Figure 10A**). To begin with, HIBCH is expressed at lower levels in tumor tissues, and high levels are associated with better clinical outcomes (**Figures 10B, C**). High levels of HIBCH expression are associated with lower tumor grades and stages, reflecting its relationship to clinical variables (**Figure 10D**). The immune microenvironment and HIBCH also appear closely linked, which classify ccRCC into 6 immune subtypes and may be useful to classify different types of ccRCC according to their immune response (**Figure 10E**). In addition to having a negative correlation with immune cell infiltration, HIBCH expression was also found to be negatively correlated with TME scores (**Figures 10F, G**). More interestingly, the immune checkpoints CTLA4 and PDCD1 showed a negative correlation with the expression level of HIBCH as well (**Figure 10H**). According to the pan-cancer analysis, HIBCH displayed similar effects in numerous cancers (SF2). Based on these findings, there may be a mechanism through which HIBCH interacts with ccRCC’s immune microenvironment, which may influence tumor development and treatment response.

**Figure 11:**
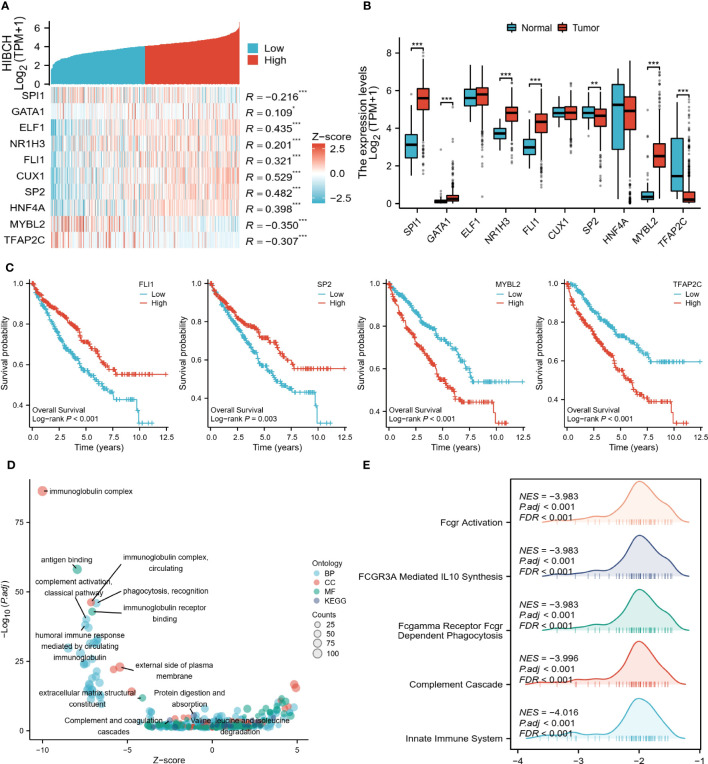
Regulatory mechanisms of HIBCH. **(A)** Heat map of HIBCH co-expression with the top 10 potential TFs. **(B)** Expression levels of potential TFs in tumor and normal tissues. **(C)** K-M survival curves of the screened TFs. **(D)** Visualized scatter plots of GO and KEGG analysis. **(E)** Visualized-mountain range plot of the top 5 of |NES| in GSEA analysis results. “*” <0.5, "**" <0.01, and "***" <0.001.

To further explore the regulatory mechanisms of HIBCH in ccRCC, we identified several potential TFs that regulate HIBCH expression, as well as potential pathways that inhibit ccRCC development. The co-expression heat map demonstrated the relationship between HIBCH expression and the top 10 TFs, most of which had high correlation coefficient values (**Figure 11A**). Difference analyses revealed significant differences between tumors and normal tissues in the expression levels of SPI1, GATA1, NR1H3, FLI1, SP2, MYBL2, and TFAP2C (**Figure 11B**). Subsequent K-M survival analysis of these DEGs revealed that FLI1, SP2, MYBL2, and TFAP2C were associated with OS (**Figure 11C**). Moreover, SP2 was lowly expressed in tumor tissues and associated with a good prognosis, while MYBL2 was highly expressed in tumor tissues and associated with a poorer prognosis. It is more likely that these two genes play a role in the regulation of HIBCH, but more experiments are needed to verify this conjecture. Following enrichment analysis, DEGs between high and low HIBCH expression groups had enriched biological functions associated with immunity (**Figure 11D**). More importantly, the GSEA results suggested that HIBCH is closely associated with FCGR-related pathways (**Figure 11E**). This gene family encodes the receptor for the Fc portion of immunoglobulin G, which is involved in a range of immune processes. This further suggests a complex mechanism of interaction between HIBCH and the immune microenvironment and has the potential to be a relevant biomarker for immunotherapeutic response.

### Experimental verification results

**Figure 12:**
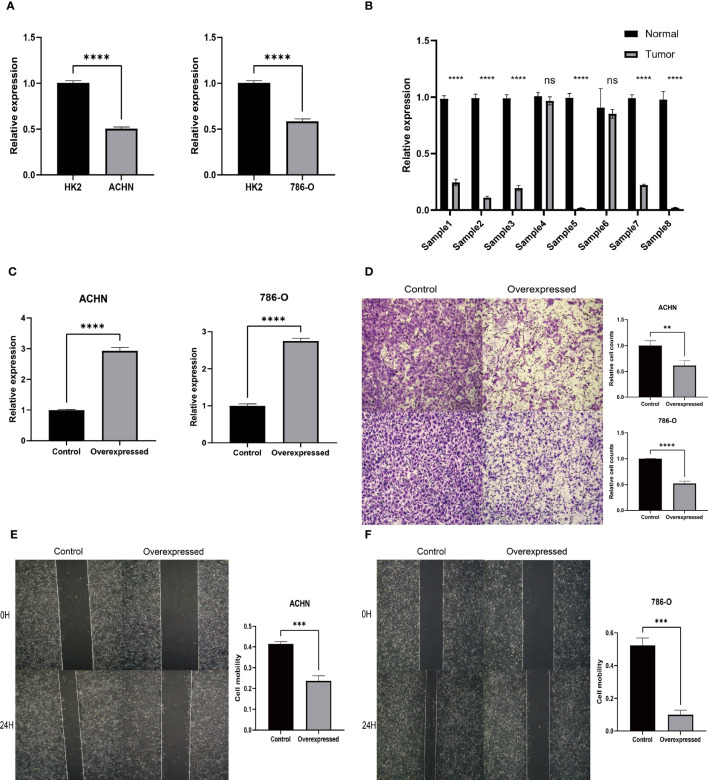
Experimental verification results. **(A)** Expression levels of HIBCH in HK-2, ACHN, and 786-O cell lines. **(B)** Differential expression of HIBCH in kidney cancer and paraneoplastic tissues. **(C)** Overexpression efficiency of HIBCH. **(D)** Transwell assay results in ACHN and 786-O cell lines. **(E, F)** Results of the scratch assay in ACHN and 786-O cell lines. "**" <0.01, "***" <0.001, and “****” <0.0001.

Our *in vitro* studies revealed that HIBCH was higher expressed in HK2 than in ACHN and 786-O (**Figure 12A**). Moreover, by extracting RNA from kidney cancer and paraneoplastic tissues for qPCR, the same results were obtained, and the cancer tissues expressed lower levels of HIBCH (**Figure 12B**). Subsequently, we further explored the effect of abnormal expression of HIBCH on the migration ability of kidney cancer cells to elucidate its role in the metastasis of kidney cancer. **Figure 12C** showed that we successfully overexpressed HIBCH in ACHN and 786-O. As compared to the overexpression group, the number of cells in the control group was significantly higher (**Figure 12D**); in the scratch assay, the control cell migration rate was also higher (**Figures 12E, F** **)**, and these results were statistically significant.

## Discussion

There are two primary mechanisms responsible for tumor occurrence and development: inactivation of tumor suppressors and activation of tumor promoters. In this process, epigenetic modifications play a key role in regulating the expression of genes (21). Current studies have shown that epigenetic aberrations are common in RCC, especially histone modifications, and are closely associated with their prognosis and treatment (22, 23). According to Zhang et al., histone lactylation modification (12) is a new epigenetic modification modality that provides new insights into the pathogenesis of RCC. Cancer is characterized by two crucial features: immune escape and metabolic reprogramming. Linking these aspects together is lactate, a metabolite that facilitates immunosuppression through lactylation modification. Recent research reveals that high levels of lactate in the tumor microenvironment (TME) hinder T cell-mediated immune responses, effectively facilitating tumor immune evasion. Additionally, histone lactylation in macrophages drives a transition toward an immunosuppressive M2 macrophage phenotype (10, 13, 24). This evidence suggests that tumor metabolism and lactylation modification can modulate each other and influence the function of immune cells in TME (25, 26). As a result, it is imperative to investigate in depth the effect of histone lactylation modifications on TME in ccRCC to predict patient survival and immunotherapeutic response.

In this study, we identified DEGs with histone lactylation modifications interfering with m6A, which were used to reveal the prognosis and TME characteristics of ccRCC. At first, the DECG obtained by screening classified ccRCC patients into two subtypes with different clinical and immunological characteristics, and then we constructed a risk model based on 11 prognostic genes. Patients at higher risk have shorter survival but had higher levels of TMB, MSI, and anti-tumor immune cell infiltration, and the easier score suggested that this group of patients was more sensitive to immune checkpoint inhibitors. The screening of patients suitable for immunotherapy is an urgent clinical problem, and our results are certainly instructive for the design of future prospective studies. The nomogram is a practical prediction tool that has important reference value in both the clinical decision-making of ccRCC and the long-term management of the disease.

As seen in our findings, patients in different risk groups have very different TME characteristics, with high-risk patients having higher immune scores. However, we also observed that the C2 subtype, which has a worse prognosis, has a lower immunization score than the C1 group, which may seem paradoxical. A Sankey diagram of the correlation between the C1/C2 subtypes and the risk grouping may explain this phenomenon, and that the C1 group, which has a slightly better prognosis, is not all distributed in the low-risk group, and that it contains a significant portion of high-risk patients. Therefore, there is some heterogeneity within the C1 group, and a more detailed delineation is needed in the future.

It is well known that the TME at which the tumor cells are located is one of the key reasons for this difference (27, 28). Various cells, stroma, and non-cellular components together constitute the TME, and not only do these components have complex interactions with each other, but they are also influenced by other factors such as metabolic and epigenetic modifications (19, 29, 30). Both lactylation modifications and m6A modifications can influence the TME, and they can not only affect the chemotaxis and activation of immune cells, but also regulate the molecules on the surface of immune cells, and thus the function of immune cells and the intensity of immune responses (31–33). The study by Jia Xiong et al. confirmed the effect of the interaction between these two epimodification modalities on the immune microenvironment (15), in other words, they may have synergistic effects in the immune microenvironment of tumors, jointly affecting tumor growth and the effectiveness of immunotherapy. For the first time, we have combined the analysis of these two epigenetic modifications for exploring the heterogeneity of ccRCC in terms of TME. More importantly, our model is not only able to accurately predict the long-term survival of patients but also has implications for immunotherapy.

Furthermore, we have identified a crucial gene in the model known as HIBCH (3-Hydroxyisobutyryl-CoA Hydrolase), which is an enzyme that plays a vital role in the metabolism of fatty acids (34). It also means that HIBCH is not only closely related to histone lactylation modification (12), but may also influence the process of mitochondrial energy metabolism. HIBCH’s role in cancer is currently unknown in the current state of research. Shan et al. delved into the implications of HIBCH in the progression and treatment of colorectal cancer. Colorectal cancers express higher levels of HIBCH, and its function depends on its localization in mitochondria, and blocking the function of HIBCH not only can inhibit the growth of cancer cells but also can improve the efficacy of targeted therapy (35). In our study, HIBCH was suggested to be reduced in expression in ccRCC and associated with a good prognosis, and the results of *in vitro* experiments also showed that HIBCH inhibits the migration ability of kidney cancer cells. To our knowledge, mitochondrial energy metabolism is not only closely related to the process of lactate metabolism (36) but also plays an important role in the progression of ccRCC (37). Therefore, we believe that HIBCH is important for finding new biomarkers in the field of ccRCC, however, more rigorous *in vitro* and *in vivo* experiments are still needed in the future to clarify the specific mechanism of HIBCH action in ccRCC.

In conclusion, our study provides a novel perspective on the prognostic significance and characteristics of the tumor microenvironment (TME) in clear cell renal cell carcinoma (ccRCC). We have developed a reliable nomogram and identified a potentially valuable biomarker. However, it is important to acknowledge certain limitations in our study. The first limitation of our study is that we rely mainly on retrospective data collected from public databases. It also means that we lacked much valuable clinical information to perform a comprehensive analysis. A second limitation is that we did not fully elucidate the specific mechanisms driving the key gene functions. While our study has limitations, it contributes to our understanding of ccRCC and provides a basis for future research.

## Conclusion

From an epigenetic standpoint, our research has uncovered distinct traits within the TME of ccRCC. Moreover, we have successfully established robust prognostic models that accurately predict patient outcomes and offer valuable insights for the effective utilization of immunotherapy. Furthermore, our data analysis and *in vitro* experiments have pinpointed a promising therapeutic target for ccRCC treatment, namely HIBCH. These findings hold great potential for advancing the field of ccRCC research and potentially improving patient outcomes.

## Data availability statement

The original contributions presented in the study are included in the article/**Supplementary Material**, further inquiries can be directed to the corresponding authors.

## Ethics statement

The studies involving humans were approved by Medical Research Ethics Committee of the First Affiliated Hospital of Nanchang University. The studies were conducted in accordance with the local legislation and institutional requirements. The human samples used in this study were acquired from primarily isolated as part of your previous study for which ethical approval was obtained. Written informed consent for participation was not required from the participants or the participants’ legal guardians/next of kin in accordance with the national legislation and institutional requirements.

## Author contributions

LY, JX, and JL: data curation, formal analysis, writing - original draft, writing - review & editing. XW: data curation, formal analysis, writing - review & editing. SL, XL, FZ, and QD: data curation, formal analysis. BF and SX: conceptualized research, writing - review & editing. All authors contributed to the article and approved the submitted version.
