# Supplementary material for: Prognostic and tumor microenvironmental feature of clear cell renal cell carcinoma revealed by m6A and lactylation modification-related genes
Source: Front Immunol. 2023 Aug 11;14:1225023. doi: 10.3389/fimmu.2023.1225023 (PMC10450969; doi:10.3389/fimmu.2023.1225023)
Supplement: Supplementary file 1 [file DataSheet_1.docx]

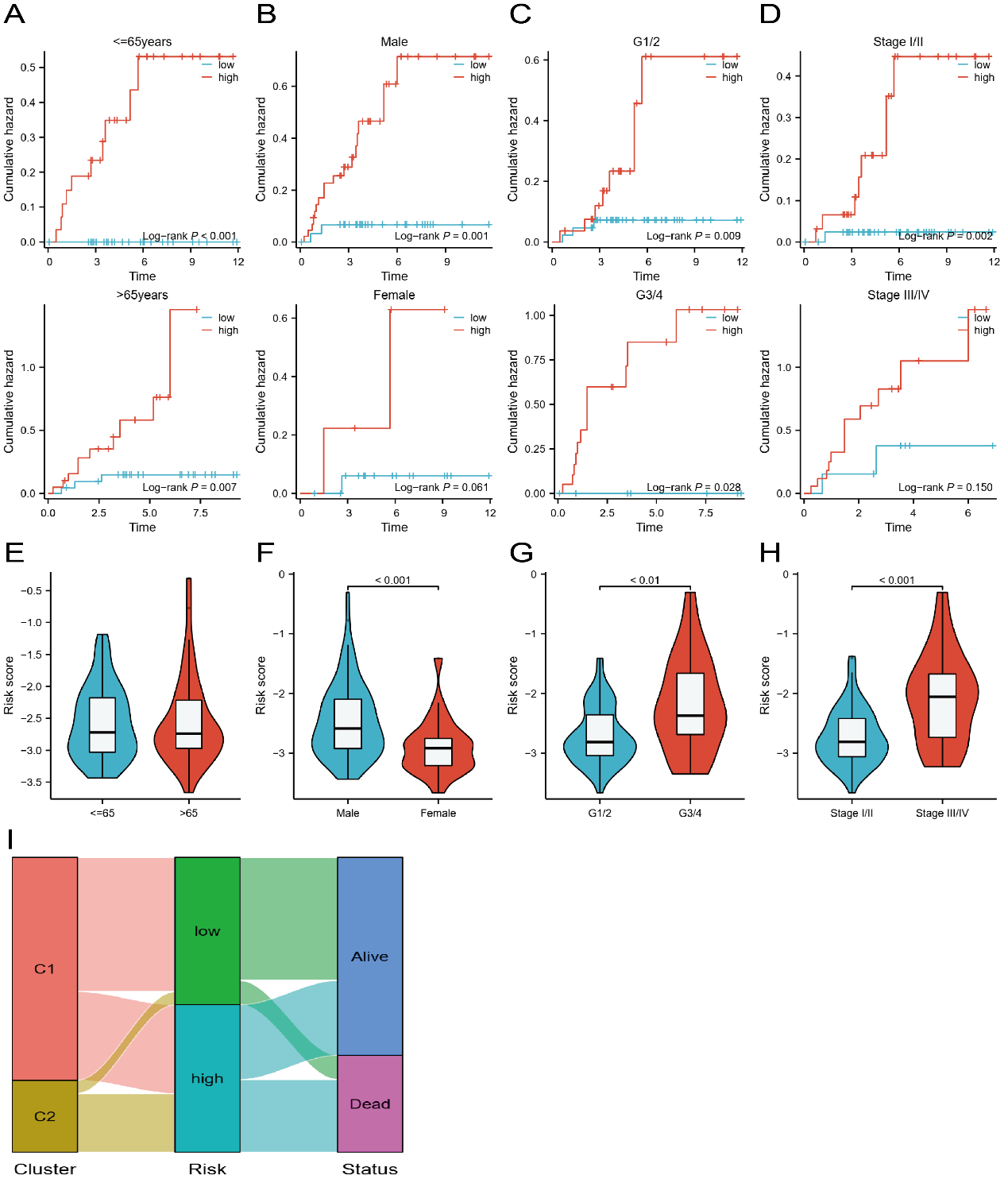


**SF1:** Risk characteristics of clinical variables by subgroup in the E-MTAB-1980 cohort. A, B, C, D: Cumulative hazard over time for <=65 and >65 years of age, male and female, G1/2 and G3/4, and Stage I/II and III/IV patients in high and low-risk groups. E, F, G, H: Differences in risk scores between patients <=65 years and >65 years, male and female patients, G1/2 and G3/4 patients, StageI/II and StageIII/IV. I: A Sankey diagram describing the correlation between C1/C2 subtypes and high or low risk.


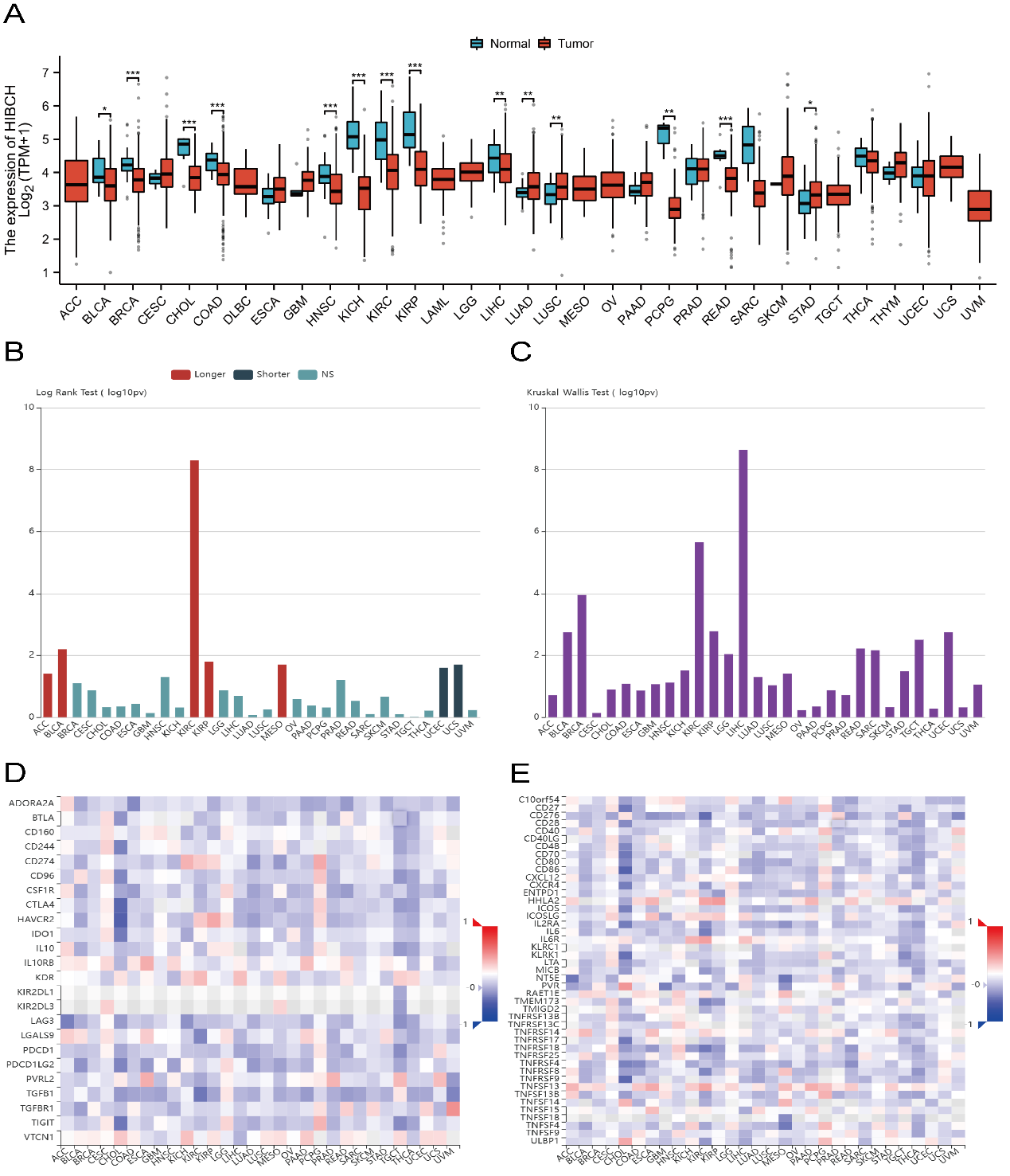


**SF2:** Results of pan-cancer analysis of HIBCH. A: Differential expression levels of HIBCH between tumors and normal tissues in various cancers. B: Survival differences (OS) of HIBCH in various cancers. C: Immunological correlation of HIBCH with various cancers. D, E: Heat map of the expression correlation between HIBCH and immunoinhibitory, and immunostimulatory.


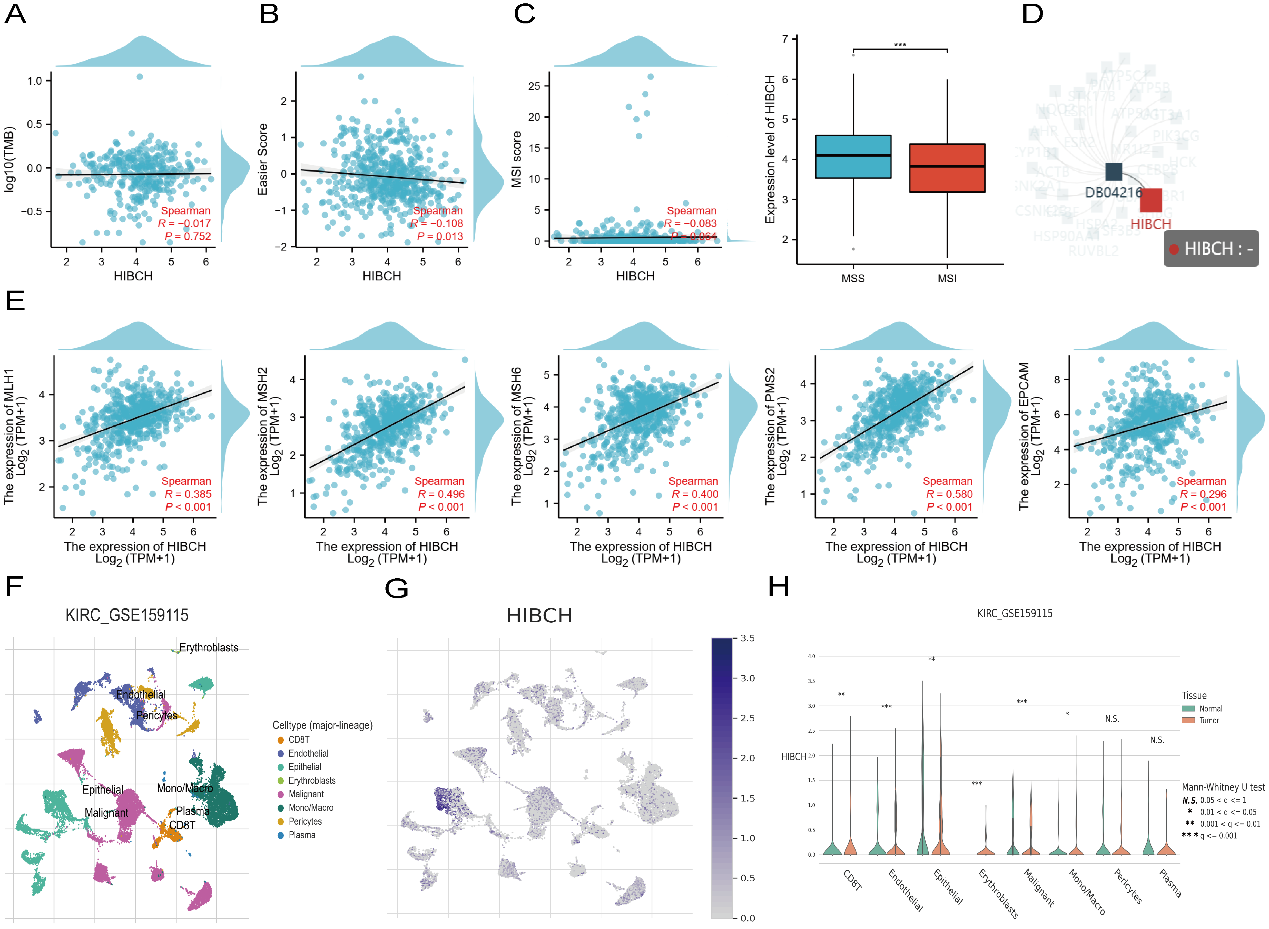


**SF3:** Immunological and single cell analysis of HIBCH. A, B, C: Correlation analysis of HIBCH with TMB, easier scores, and MSI scores. D: Potential therapeutic agent DB04216 (Quercetin) for targeting HIBCH. E: Correlation analysis of HIBCH with MSI-related DNA mismatch repair genes. F: Annotated cell type map of the results of single cell analysis of the GSE159115 cohort. G: Distribution map of HIBCH in each cell type. H: Differences in the expression levels of HIBCH in each cell type.
